# Efficacy and safety of neoadjuvant chemoradiotherapy combined with immunotherapy for locally advanced esophagogastric junction or gastric cancer: a systematic review and meta analysis

**DOI:** 10.3389/fimmu.2026.1745356

**Published:** 2026-05-29

**Authors:** Xiaoxuan Ma, Wei Guo, Jialin Sun, Yanyan Liang, Chuan Yao, Yuteng Chi, Zifeng Chi, Teng Li, Ruifeng Wang, Ruiyang Gao, Yameng Gao, Ziteng Zhang, Mingchang Miao, Kai Shang, Chao Gao

**Affiliations:** 1 Department of Radiation Oncology,The Fourth Hospital of Hebei Medical University, Shijiazhuang, China; 2 Hebei Medical University, Shijiazhuang, China; 3 Department of Ophthalmology, The First Hospital of Hebei Medical University, Shijiazhuang, China; 4 The Fourth Hospital of Hebei Medical University, Shijiazhuang, China

**Keywords:** gastroesophageal junction orgastric cancer (EGJ/GC), neoadjuvant chemoradiotherapy, neoadjuvant immunotherapy, PD-1/PD-L1 inhibitors, systematic review

## Abstract

**Background:**

With significant progress made in immunotherapy for locally advanced and metastatic gastroesophageal junction or gastric cancer (EGJ/GC), multiple studies have been initiated on neoadjuvant chemoradiotherapy (nCRT) combined with immune checkpoint inhibitors (nCRT+ICIs) for locally resectable EGJ/GC. Consequently, current clinical trials investigating nCRT+ICIs for locally advanced EGJ/GC were summarized within this study. A systematic review and meta-analysis were performed to evaluate the efficacy and safety of this combination therapy, with the objective of providing clinicians with robust, evidence-based treatment strategies and clinical references.

**Materials and methods:**

Relevant studies were retrieved from electronic databases including PubMed, Embase, the Cochrane Library, ClinicalTrials.gov, ASCO, ESMO, and CNKI. Efficacy was evaluated based on the pathological complete response (pCR) rate, major pathological response (MPR) rate, downstaging and the R0 resection rate. Safety was assessed by the incidence of grade ≥3 treatment-related adverse events (TRAESs) and grade ≥3 immune-related adverse events (irAEs). All statistical analyses were performed using Stata version 15.0.

**Results:**

A total of 12 studies fulfilled the inclusion criteria for analysis. The pooled rates of pathological complete response (pCR), major pathological response (MPR), and R0 resection were found to be 29%, 52%, and 100%, respectively. The pooled T-stage downgrading rate was 51%. The N-stage downstaging rate was 73%, with all cases successfully downstaged to ypN0. The incidences of grade ≥3 TRAESs and irAEs were 47% and 6%, respectively. The pooled 2-year progression-free survival (PFS) rate was 65%, while the 1-year and 2-year overall survival (OS) rates were 91% and 74%, respectively. Subgroup analyses indicated pCR rates of 26% and 33% for the programmed death-ligand 1 (PD-L1) and programmed death-1 (PD-1) inhibitor subgroups, respectively. Comparable pCR rates of 29% were observed for both sequential and concurrent chemoradiotherapy combined with immunotherapy. Within the concurrent therapy subgroup, a higher pCR rate was achieved with PD-1 inhibitor-based regimens (36%) than with PD-L1 inhibitor-based regimens (25%), while a 29% pCR rate was demonstrated for PD-L1 inhibitor sequential therapy. Stratification by the PD-L1 combined positive score (CPS) yielded pCR rates of 22% for CPS <1, 23% for CPS 1–5, and 51% for CPS ≥5. Radiation dose stratification showed pooled pCR rates of 26% for doses <45Gy and 33% for doses ≥45Gy. Regional variations were observed, with Asian cohorts exhibiting a higher pCR rate (36%) than Western cohorts (26%).

**Conclusion:**

In locally advanced EGJ/GC, the combination of PD-1/PD-L1 inhibitors with nCRT demonstrates promising response rates and acceptable toxicity profiles. Notably, this regimen achieved pCR rate of 51% in patients with CPS ≥5. Furthermore, favorable outcomes were observed even in those with low CPS. This study is primarily based on phase II or single-arm data; therefore, these results remain preliminary. These findings require further validation in future large-scale randomized controlled trials (RCTs).

**Systematic review registration:**

https://www.crd.york.ac.uk/prospero/, identifier CRD42024590688.

## 1 Introduction

Gastroesophageal junction or gastric cancer (EGJ/GC) often presents with nonspecific and insidious symptoms, frequently resulting in a diagnosis at locally advanced stages in most patients. Radical surgery remains the standard of care EGJ/GC. However, despite D2 radical gastrectomy followed by adjuvant therapy, high recurrence rates (50%–80%) and suboptimal 5-year survival rates (10%–33%) are still observed. In this context, neoadjuvant therapy has been widely recognized as an important strategy aimed at reducing tumor burden before surgery, evaluating tumor response, lowering the postoperative recurrence rate, improving local control rate, and ultimately enhancing overall survival (OS) in patients.

Multiple studies have established the pivotal role of neoadjuvant chemoradiotherapy (nCRT) in the management of locally advanced resectable EGJ/GC, demonstrating the efficacy of preoperative chemoradiation and its potential survival benefits (1–3). However, the advent of immune checkpoint inhibitors (ICIs) targeting programmed death-ligand 1 (PD-L1) or programmed death-1 (PD-1) pathway has ushered in a new era of immunotherapy for EGJ/GC. Preclinical evidence has demonstrated that synergistic effects are exerted by that nCRT+ICIs exert synergistic effects through multiple biological mechanisms: chemoradiation induces immunogenic cell death (ICD) facilitating the release of tumor antigens and the upregulation of MHC-I expression on tumor cells. This process enhances antigen presentation while remodeling the tumor microenvironment (TME) into an immunologically “hot” state that promotes T-cell infiltration and activation (4, 5). Concurrently, PD-L1 expression is upregulated by radiation, whereas antitumor activity is enhanced by ICIs through the blockade of PD-1/PD-L1 signaling to alleviate T-cell exhaustion. (6). Moreover, the combination of radiotherapy and ICIs can induce abscopal effects (7).

Based on the aforementioned mechanisms, numerous clinical trials have been conducted worldwide to explore the value of nCRT+ICIs in locally advanced EGJ and gastric cancer (8–19). These trials have yielded impressive results, highlighting the potential of nCRT+ICIs. Although the potential of nCRT+ICIs has been demonstrated in multiple clinical trials, controversies persist regarding the underlying efficacy mechanisms, optimal regimens, safety profiles, and individualized patient selection. Therefore, the efficacy and safety of nCRT+ICIs for locally advanced EGJ/GC were evaluated in this meta-analysis, with the objective of identifying the optimal treatment strategy.

## 2 Methods

### 2.1 Search strategy

We conducted a comprehensive search of multiple online databases, including PubMed, Embase, the Cochrane Library, ClinicalTrials.gov, ASCO, ESMO, and CNKI. An additional electronic search was performed by thoroughly reviewing conference proceedings from two premier international meetings: the Annual Meeting of the American Society of Clinical Oncology (ASCO) and the European Society for Medical Oncology (ESMO) Congress, to account for potentially unpublished findings. The last search was updated to January 2025. The search strategy was: “Stomach Neoplasms” (MeSH Terms), “Esophagogastric Junction” (MeSH Terms), and “Immune Checkpoint Inhibitors” (All Fields). Furthermore, we manually screened reference lists of included studies and relevant reviews to ensure no eligible studies were overlooked. The study protocol has been registered in the PROSPERO database (Registration ID: CRD42024590688).

### 2.2 Inclusion and exclusion criteria

Inclusion criteria:

1. Participants with resectable locally advanced (cT3/4aN+M0) esophagogastric junction or gastric cancer (EGJ/GC); EGJ tumors were defined as Siewert types I–III, including distal esophageal cancers consistent with Siewert type I.
2. Intervention: neoadjuvant chemoradiotherapy combined with immunotherapy;
3. Study designs: randomized controlled trials, single-arm studies, or prospective cohort studies;
4. Publications must include detailed treatment protocols and report at least one of the following key outcomes: pathological complete response (pCR) rate, major pathological response (MPR) rate, R0 resection rate, downstaging rate or rate of grade≥3 treatment-related adverse events (TRAEs) incidence (with grading performed according to CTCAE v5.0 criteria), where the data are either directly reported or can be indirectly derived from the study.

Exclusion criteria:

1. Patients with distant metastatic or unresectable tumors;
2. Studies without available data;
3. Publication types: reviews, commentaries, retrospective analyses, case reports, and cellular or animal studies.

### 2.3 Quality assessment

Most of the included trials were single-arm studies. Consequently, the studies were categorized as either “poor” (0–12 points) or “good” (13–16 points) based on the MINORS score, listed in that order. Any discrepancies were resolved through consensus discussion.

### 2.4 Data extraction

Two investigators independently evaluated the data from eligible studies, and the key information extracted from the original studies included:

1. Study characteristics: first author, country, publication year, study type, and follow-up time.
2. Patient baseline: number of patients, age, gender, and tumor stage.
3. Treatment information: dose and fraction of radiation therapy, the sequence and regimens of chemotherapy and immunotherapy drugs and adjuvant therapy.
4. Study outcomes: R0 resection rate, pCR rate, MPR rate, downstaging rate, OS, disease-free survival (DFS), progression-free survival (PFS), and rate of grade ≥ 3 AEs.

### 2.5 Statistical analysis

All statistical analyses were performed using STATA/SE version 15. A random-effects model was employed, with study heterogeneity classified as low (I² < 50%) or high (I² > 50%) using Cochran’s Q chi-square test and I² statistics. Sensitivity analyses were conducted by sequentially excluding studies contributing to substantial heterogeneity in the pooled data. To stabilize the variance of binomial proportions, the Freeman-Tukey double arcsine transformation was applied before pooling the data, with the final results back-transformed to the original scale for clinical interpretation.

## 3 Results

### 3.1 Identification of studies

**Figure 1:**
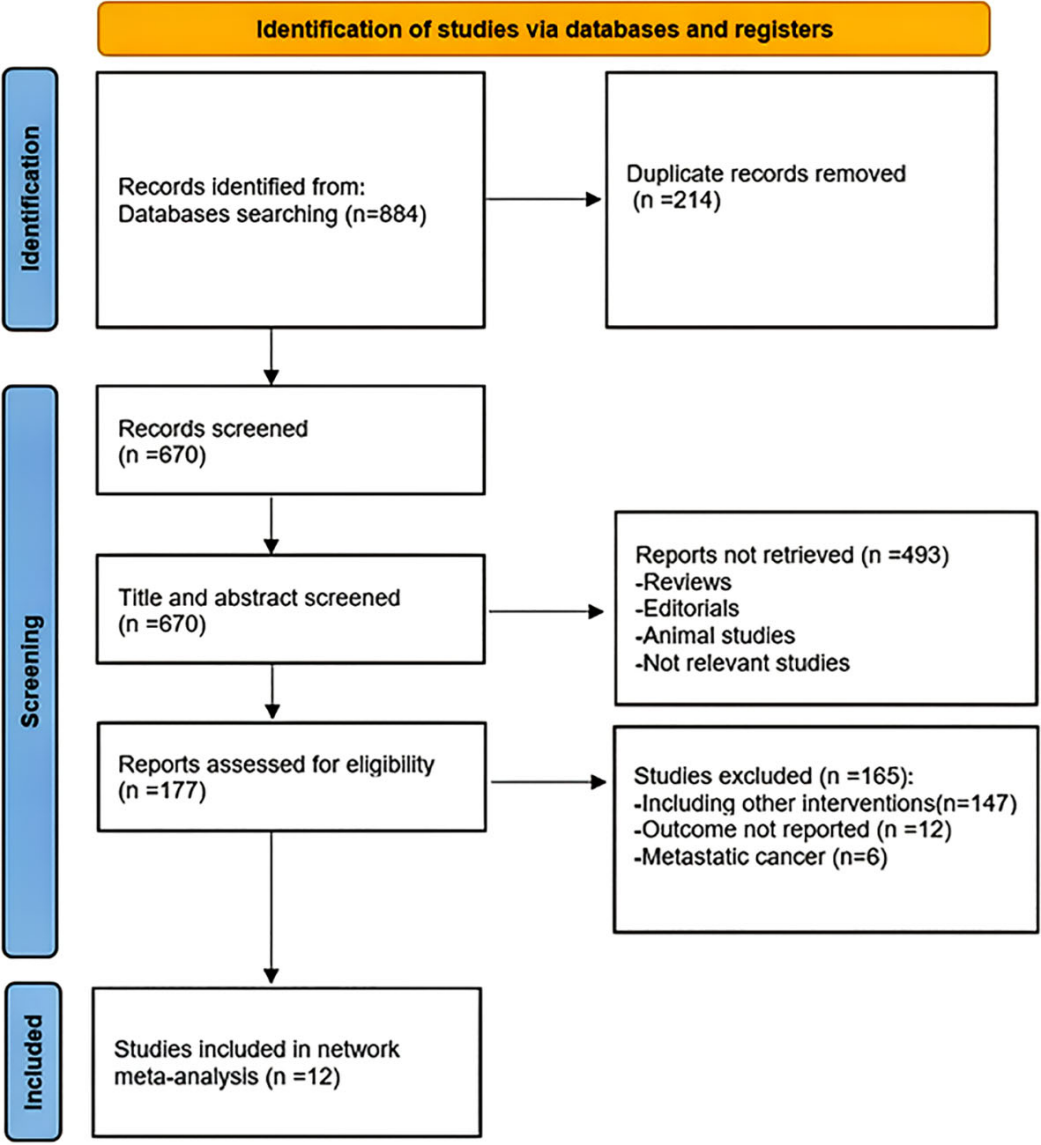
The flow diagram of this meta-analysis.

As illustrated in **Figure 1**, a final total of 12 studies was included following the screening of titles and abstracts, the removal of duplicates, and the performance of full-text reviews. All 12 studies were rated as being of “good” quality according to the MINORS scoring system, with findings summarized in **Table 1**.

**Table 1:** Characteristic of included studies.

Author year	NCT number	TYPE	No. of patient	Age	Gender	Clinical T stage	Clinical N stage	Primary tumor location	PD-L1 CPS	Her2	Intervention	Radiotherapy dose	MINORS score
Uboha et al., 2025	NCT03490292	Single arm	22	64 (42–76)	Male 20 Female 2	T2: 1(5%) T3: 18(95%)	N0 : 7(37%) N+: 12(63%)	E: 8(36%) GEJ: 14(64%)	all<5	–	PC+radiotherapy+Avelumab→ surgery→Avelumab	41.4Gy/23f	13
Schloesser et al., 2023	NCT04159974	Single arm	56	–	–	–	–	GEJ: 56(100%)	–	–	PC+radiotherapy+Durvalumab→surgery	41.4Gy/23f	14
Du et al., 2022	NCT04061928	Single arm	24	–	–	–	–	GEJ: 24(100%)	–	–	SOX+radiotherapy+ Toripalimab→surgery→SOX+ Toripalimab	45Gy/25f	13
Kelly et al., 2024	NCT03044613	RCT	A:16 B:16	A: 61 (39-73) B:66 (57-72)	A: Male 13 Female 3 B: Male 13 Female 3	–	N0 : 8(25%) N+: 24(75%)	distal E: 26 (81%) GEJ: 6(19%)	<1: 11 1–4: 5 5-10: 4 >10: 9	–	Arm A: Nivolumab→ PC+radiotherapy→surgery Arm B: Nivolumab +relatlimab→ PP+radiotherapy →surgery	41.4Gy/23f	13
Cowzer et al., 2023	NCT02962063	Single arm	36	62	Male 30 Female 6	T1:1 (2.8%) T2:2 (5.6%) T3:30 (83.3%) T4:3 (8.3%)	N0: 13(36.1%) N1: 20(55.6%) N2: 3(8.3%)	E: 11 (30%) GEJ: 25 (70%)	<1: 11 1–4: 10 ≥5: 14	Negative 30 Positive 3 Unknown 3	mFOLFOX6→durvalumab→ radiotherapy+ Oxaliplatin+5FU/Capecitabine or PC→surgery→ durvalumab	50.4Gy/28f	13
Zhu et al., 2022	NCT02730546	Single arm	31	62 (44-76)	Male 30 Female 1	T2:2 (6.5%) T3:29 (93.5%)	N0: 4(12.9%) N1: 13(41.9%) N2: 12(38.7%) N3: 2(6.5%)	GEJ: 29 (93%) G: 2 (7%)	<1 :11 1–9 :11 ≥10: 8	–	pembrolizumab→ PC+radiotherapy→surgery →pembrolizumab	41.4Gy/23f	15
Tang et al., 2022	NCT03631615	Single arm	36	65.5 (35-72)	Male 28 Female 8	T3:6 (16.7) T4a:30 (83.3)	N+: 36(100%)	GEJ: 19(53%) G: 17(47%)	<1: 16 1-4: 9 5-10: 8 ≥10: 7	–	XELOX+ Camrelizumab+radiotherapy→Surgery →XELOX	45Gy/25f	15
Wei et al., 2023	ChiCTR1900024428	Single arm	34	65.5 (58–68)	Male 28 Female 6	T3:10 (29.4%) T4a:19(55.9%) T4b:5 (14.7%)	N1: 1(2.9%) N2: 21(61.8%) N3: 12(35.3%)	GEJ: 31(91%) G: 3 (9%)	<1: 16 1-5: 5 ≥5 11	0 19 1+ 10 2+ 5	nab-paclitaxel + S-1+Sintilimab+radiotherapy →surgery	45Gy/25f	14
Roy et al., 2024	ACTRN12619000288123	Single arm	48	65 (44-81)	Male 45 Female 3	–	–	GEJ: 48(100%)	–	–	PC+radiotherapy+Avelumab→ surgery	41.4Gy/23f	13
van den Ende et al., 2021	NCT03087864	Single arm	40	63 (40-75)	Male 35 Female 5	T2:10 (25%) T3:29 (72.5%) T4a:1 (2.5%)	N0: 13(32.5%) N1: 18(45%) N2: 9(22.5%)	Mid E: 2(5%) distal E: 31 (77%) GEJ: 7(18%)	CPS<1 26 ≥10 17 ≥25 15	–	PC+radiotherapy+ Atezolizumab→surgery	41.4Gy/23f	13
Ku et al., 2024	NCT02962063	Single arm	16	–	–	≥T3: 16(100%)	N+: 16(100%)	–	–	–	mFOLFOX6→durvalumab and tremelimumab →radiotherapy+Oxaliplatin+ 5FU/Capecitabine or PC→surgery→durvalumab and tremelimumab	41.4Gy/23f	13
Shah et al., 2021	NCT02998268	Single arm	31	68 (38-81)	–	–	–	GEJ: 31(100%)	–	–	PC±pembrolizumab→ radiotherapy+Pembrolizumab→surgery→pembrolizumab	41.4Gy/23f	13

pCR pathologic complete response, MPR major pathologic response, MINORS methodological index for non-randomized studies. E: Esophagus. GEJ: Gastroesophageal Junction. G: Gastric. PC: paclitaxel and carboplati. S-1: Tegafur.

### 3.2 Efficacy

#### 3.2.1 Tumor response

**Figure 2:**
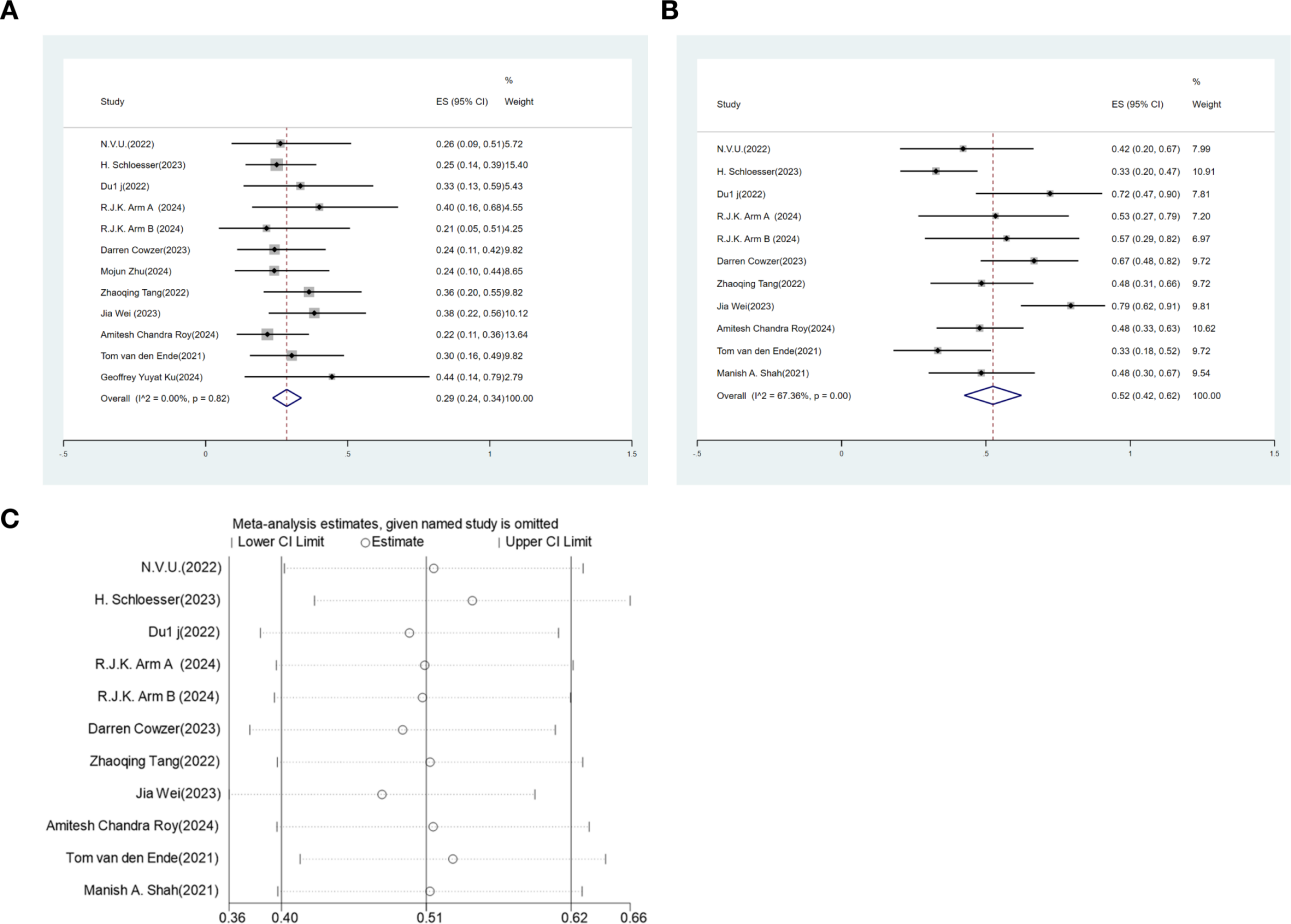
**(A)** Forest plots of pathological complete response rate. **(B)** Forest plots of major pathological response rate. **(C)** Sensitivity analysis of major pathological response rate.

Data regarding pCR from 11 studies involving 335 patients were analyzed. A pooled pCR rate of 29% (95% CI: 24%–34%) was identified, with no observed heterogeneity (I² = 0%, P = 0.82) (**Figure 2A**). Outcomes for MPR were reported by a total of 10 studies enrolling 328 patients; the pooled MPR rate was 52% (95% CI: 42%–62%), although substantial heterogeneity was observed (I² = 67.36%, P < 0.001) (**Figure 2B**). The stability of these meta-analytic findings for MPR rates was confirmed through sensitivity analysis (**Figure 2C**).

#### 3.2.2 R0 resection rate

**Figure 3:**
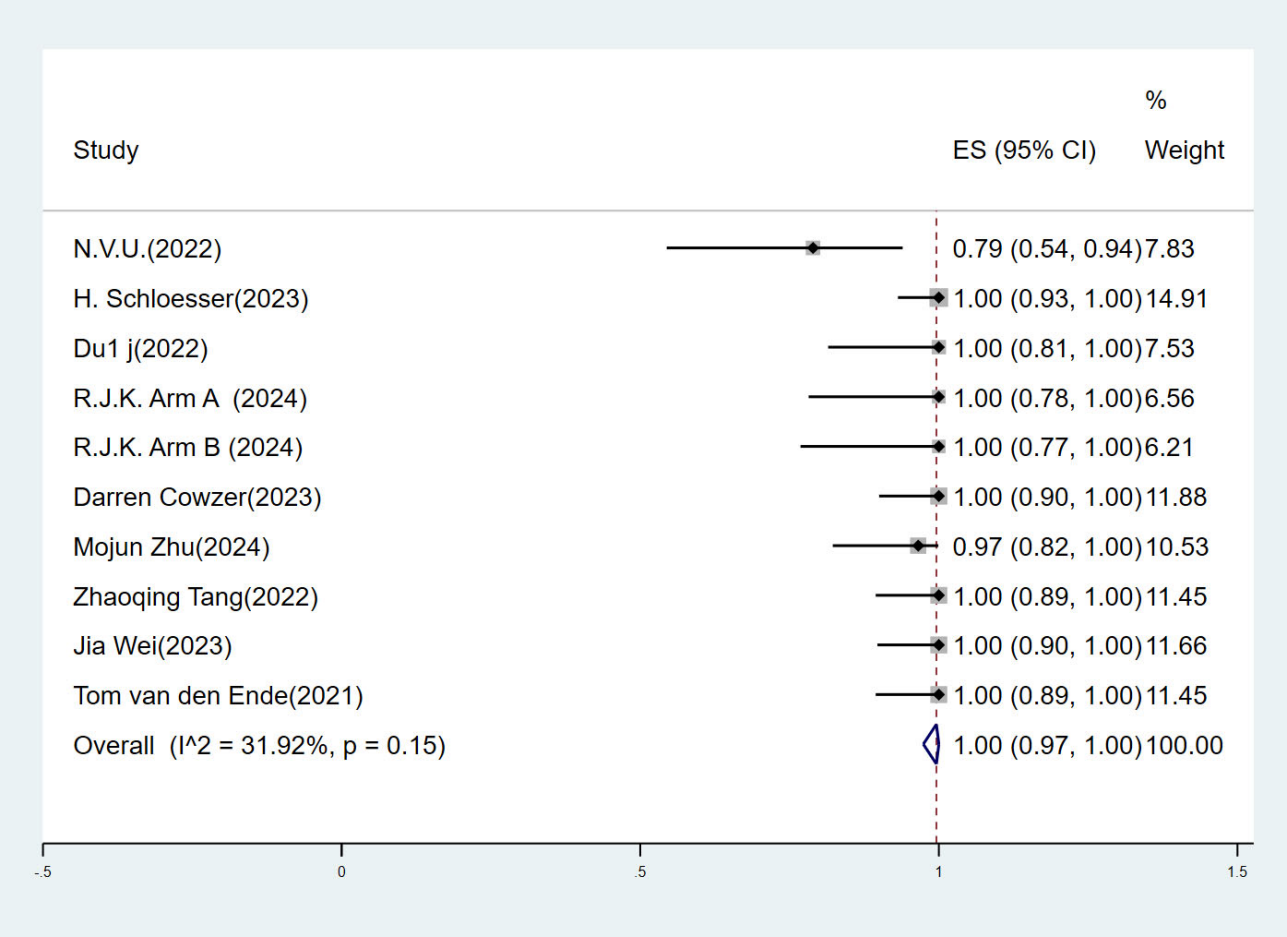
R0 resection rate.

An R0 resection rate of 100% (95% CI: 97%–100%) was demonstrated by the pooled analysis of 10 studies (enrolling 278 patients), accompanied by negligible heterogeneity (I² = 31.60%, p = 0.16) (**Figure 3**).

#### 3.2.3 Downstage

**Figure 4:**
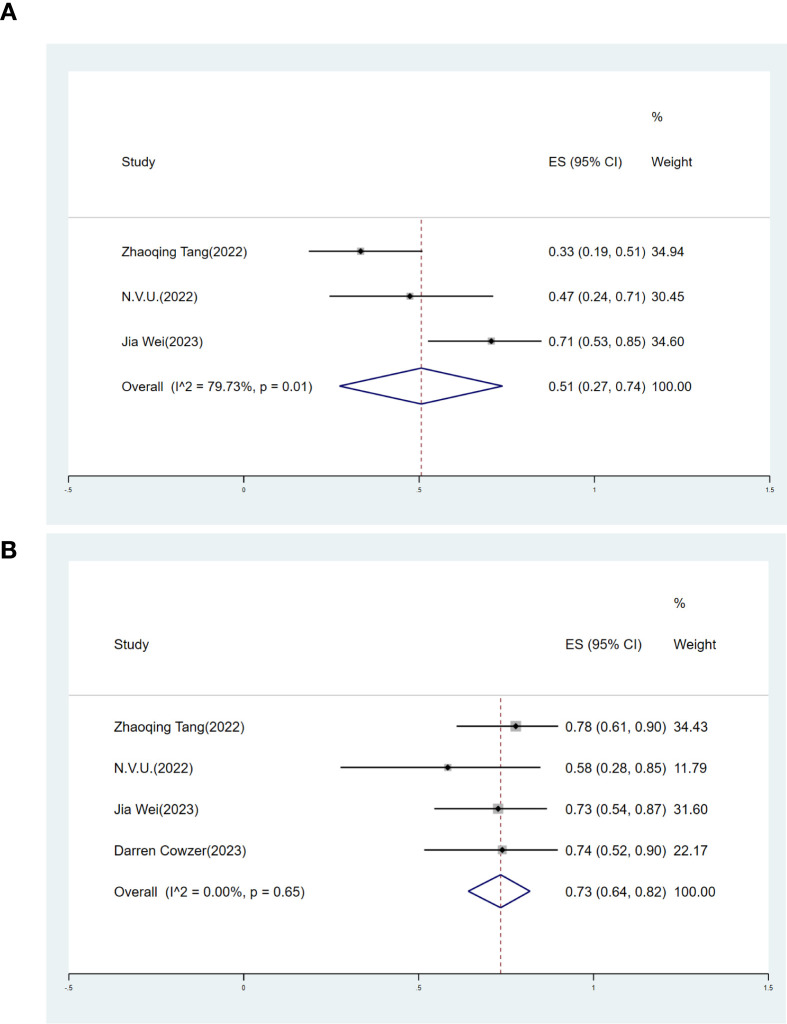
**(A)** The pooled T downstaging rate. **(B)** The pooled N downstaging rate.

Data concerning tumor downstaging were extracted and analyzed from four studies. Among them, postoperative T-category downstaging was achieved in 89 patients, while N-category downstaging was observed in 104 patients. The pooled T-stage downstaging rate was calculated as 51% (95% CI: 27%–74%; I² = 79.73%; P = 0.01) (**Figure 4A**). Within the pooled analysis, lymph node downstaging was achieved by 73% of patients (95% CI: 64%–82%; I² = 0.00%; P = 0.65), all of whom demonstrated complete nodal regression to ypN0 (**Figure 4B**).

#### 3.2.4 Survival

**Figure 5:**
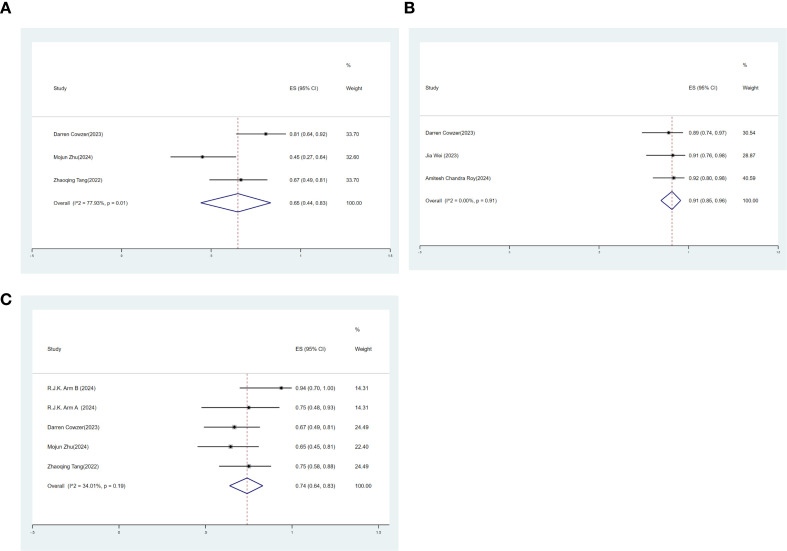
**(A)** The pooled 2-year PFS rate. **(B)** The pooled 1-year OS rate. **(C)** The pooled 2-year OS rate.

A 2-year PFS rate of 65% (95% CI: 44%–83%; I² = 77.93%, P = 0.01) was demonstrated through the pooled analysis of three studies (enrolling 101 patients) (**Figure 5A**). A pooled 1-year OS rate of 91% (95% CI: 85%–96%; I^2^ = 0%, P = 0.91) was yielded by the analysis of three studies (enrolling 118 patients) (**Figure 5B**), while five studies (enrolling 139 patients) revealed a pooled 2-year OS rate of 74% (95% CI: 64%–83%; I² = 34.01%, P = 0.19) (**Figure 5C**). Survival outcomes of the included studies in **Table 2**.

**Table 2:** Survival results of included studies.

Study	Median follow-up, months	Median OS, months	Median DFS, months	Median PFS, months	1y DFS	2y DFS	1y PFS	2y PFS	1y OS	2y OS
Ku et al., 2024 ArmA	43.9	NA	34.1	NA	NA	NA	NA	NA	NA	75%
Ku et al., 2024 ArmB	27.5	NA	NA	NA	NA	NA	NA	NA	NA	93.8%
Cowzer et al., 2023	25.5	NA	NA	NA	NA	NA	81%	71%	92%	85%
Zhu et al., 2022	24.2	NA	NA	19.6	NA	NA	NA	48.5%	NA	66.3%
Tang et al., 2022	26.3	NA	NA	NA	NA	NA	NA	66.9%	NA	76.1%
Wei et al., 2023	18.2	NA	17.0	NA	64.5%	NA	NA	NA	92.6%	NA
Roy et al., 2024	NA	NA	NA	NA	83.8%	NA	NA	NA	91.2%	NA

### 3.3 Safety

**Figure 6:**
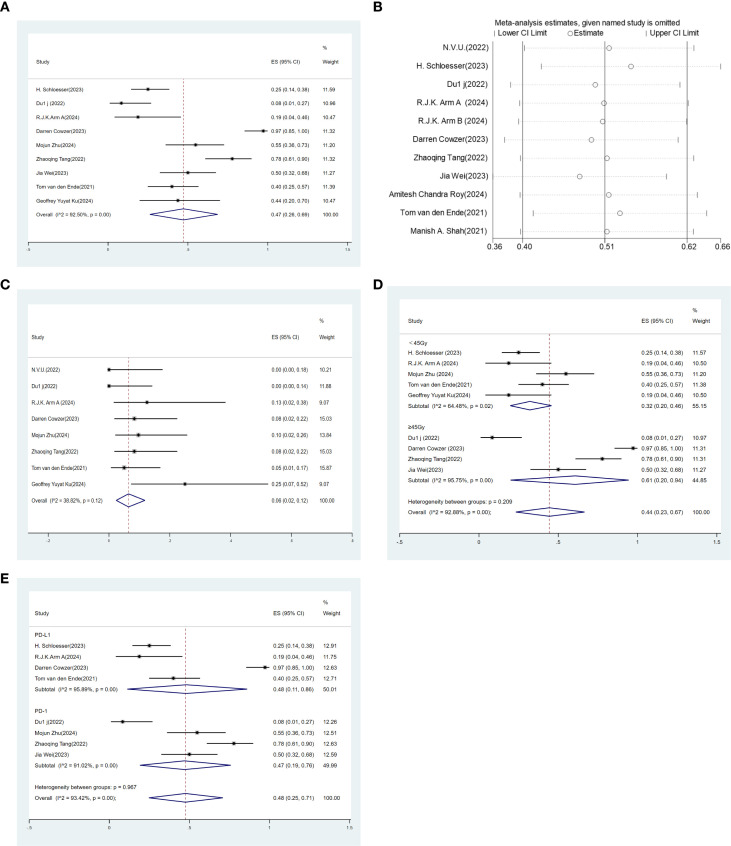
**(A)** Forest plots of grade≥3 AEs. **(B)** Sensitivity analysis of grade≥3 AEs. **(C)** Forest plots of immune-related grade≥3 AE. **(D)** Forest plots of Radiotherapy dose based on grade≥3 AEs. **(E)** Forest plots of PD-1/PD-L1 inhibitors based on grade≥3 AEs.

The pooled incidence of grade ≥3 TRAESs associated with immunotherapy combined with nCRT was found to be 47% (95% CI: 26%–69%) based on an analysis of nine trials (involving 289 patients)with substantial heterogeneity (I² = 92.50%, P < 0.001) (**Figure 6A**). It was demonstrated through sensitivity analysis that the overall meta-analysis results were not significantly influenced by any single study, thereby indicating the robust stability of these findings (**Figure 6B**). The pooled incidence of grade ≥3 immune-related adverse events(irAEs) was 6% (95% CI: 2%–12%; I² = 38.82%, P = 0.12) (**Figure 6C**). Regarding radiotherapy dosage, pooled incidences of grade ≥3 adverse events were 32% (95% CI: 20% –46%; I² = 64.48%, P = 0.03) for doses <45 Gy and 61% (95% CI: 20%–94%; I² = 95.75%, P < 0.001) for doses ≥45 Gy, respectively (**Figure 6D**). When stratified by inhibitor type, the incidence rates were 48% (95% CI: 11%–86%, I² = 95.89%, P < 0.001) for PD-L1 inhibitors and 47% (95% CI: 19%–76%, I² = 91.02%, P < 0.001) for PD-1 inhibitors (**Figure 6E**).

### 3.4 Subgroup analysis

#### 3.4.1 Subgroup based on PD-1/PD-L1 inhibitors

**Figure 7:**
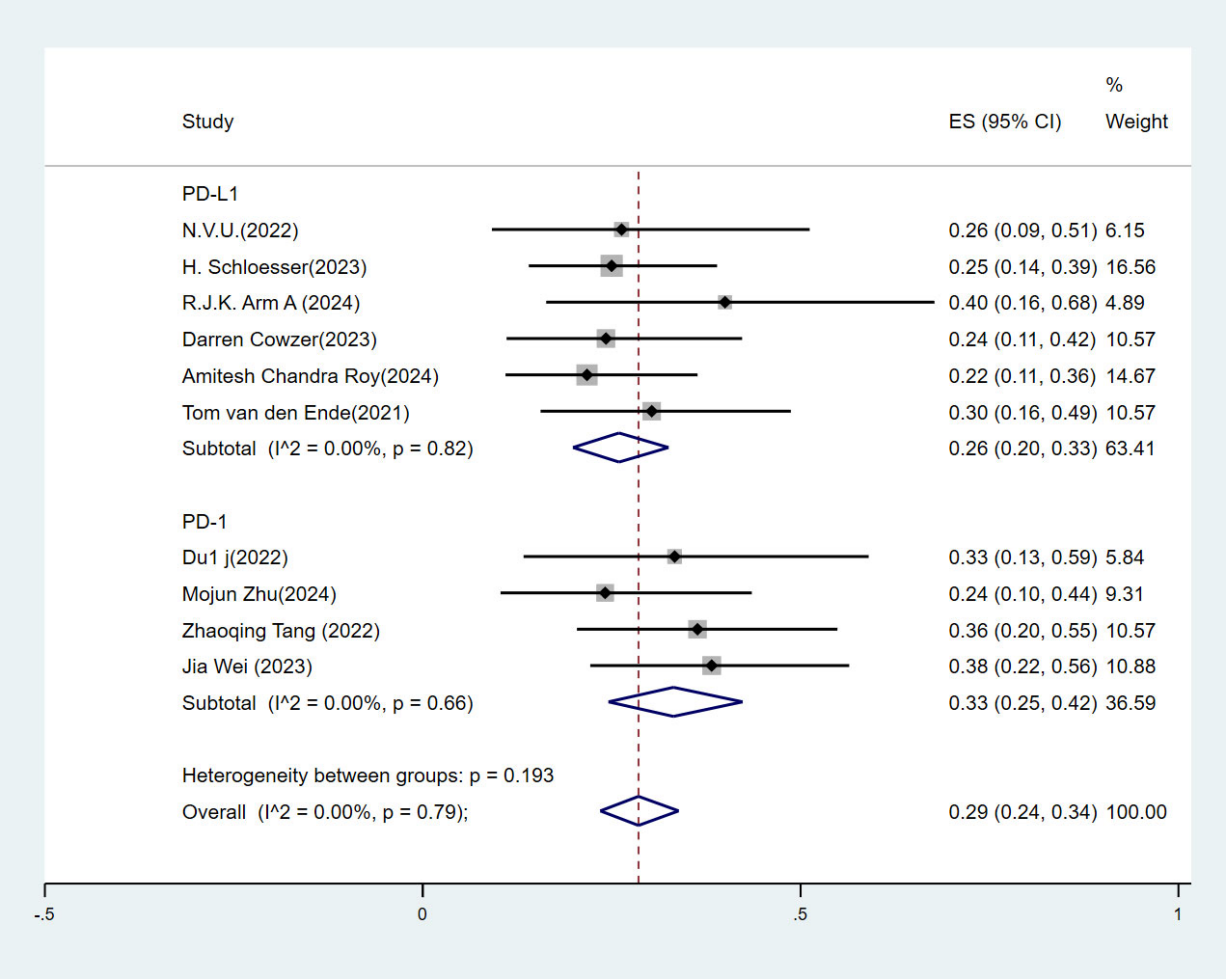
Forest plots of PD-1/PD-L1 inhibitors based on pathological complete response rate.

The pCR rates were 26% (95% CI: 20%–33%, I² = 0%, P = 0.82) in the PD-L1 inhibitor subgroup and 33% (95% CI: 25%–42%, I² = 0%, P = 0.66) in the PD-1 inhibitor subgroup (**Figure 7**).

#### 3.4.2 Subgroup based on neoadjuvant therapy strategies

**Figure 8:**
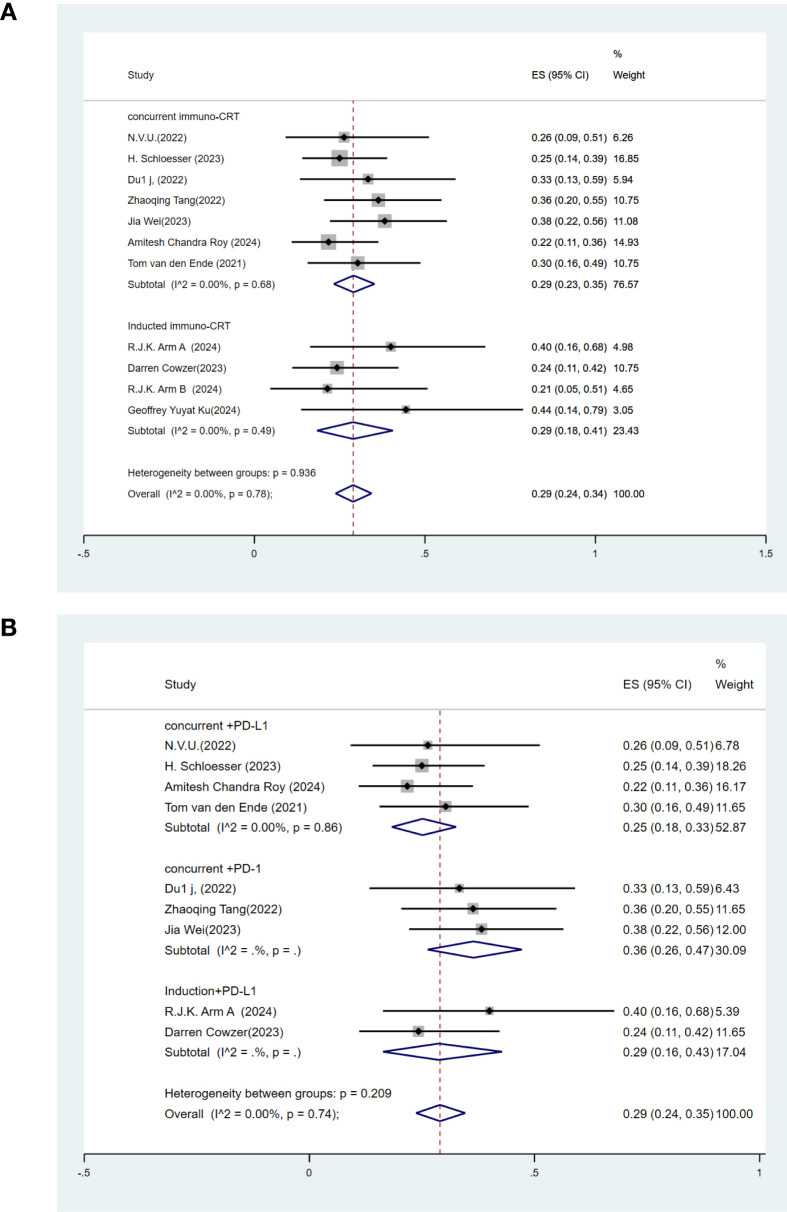
**(A)** Forest plots of treatment sequence based on pathological complete response rate. **(B)** Forest plots of treatment sequence and PD-1/PD-L1 inhibitors based on pathological complete response rate.

pCR rates of 29% (95% CI: 23% –35%; I² = 0%, P = 0.68) and 29% (95% CI: 18% –41%; I² = 0%, P = 0.49) were identified for the concurrent and sequential subgroups, respectively, via pooled analysis (**Figure 8A**). A pCR rate of 36% (95% CI: 26%–47%) was achieved with concurrent therapy combined with PD-1 inhibitors, which was notably higher than the rates observed for concurrent therapy with PD-L1 inhibitors (25%) and sequential therapy with PD-L1 inhibitors (29%) (**Figure 8B**).

#### 3.4.3 Subgroup based on different PD-L1 CPS values

**Figure 9:**
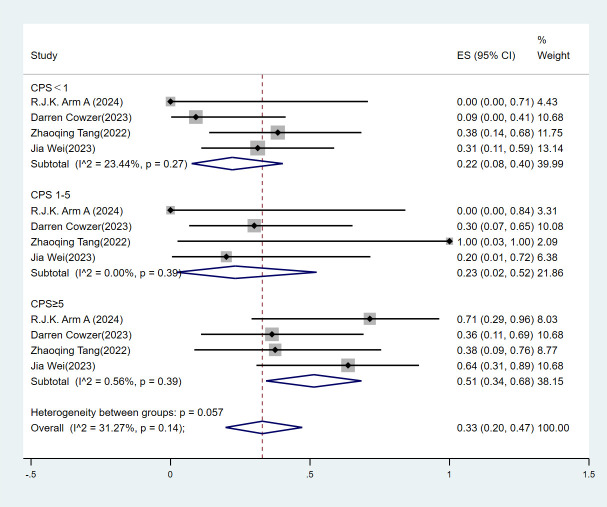
Forest plots of different PD-L1 CPS values based on pathological complete response rate.

Patients were stratified into three subgroups based on the Combined Positive Score (CPS) (<1, 1-5, and ≥5), yielding pCR rates of 22% (95% CI: 8%–40%; I²=23.44%, P = 0.27), 23% (95% CI: 2%–52%; I²=0%, P = 0.39), and 51% (95% CI: 34%–68%; I²=56%, P = 0.39), respectively (**Figure 9**).

#### 3.4.4 Subgroup based on radiotherapy dose

**Figure 10:**
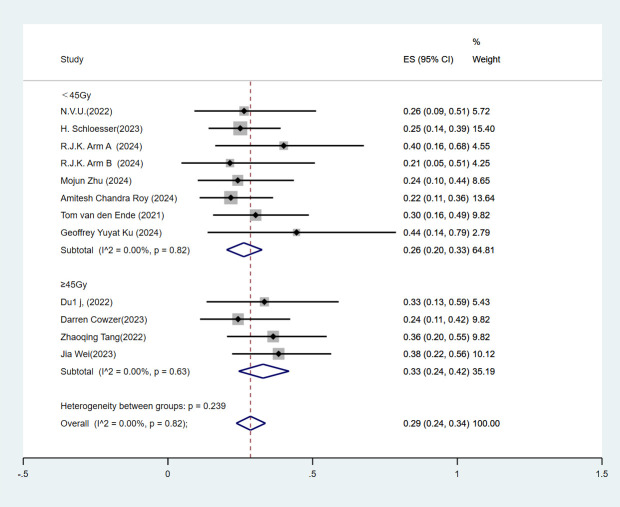
Forest plots of radiotherapy dose based on pathological complete response rate.

The radiation dose-based subgroup analysis demonstrated pooled pCR rates of 26% (95% CI: 20%-33%; I²=0%, P = 0.82) for the <45Gy subgroup and 33% (95% CI: 24%-42%; I²=0%, P = 0.63) for the ≥45Gy subgroup (**Figure 10**).

#### 3.4.5 Subgroup based on regional differences

**Figure 11:**
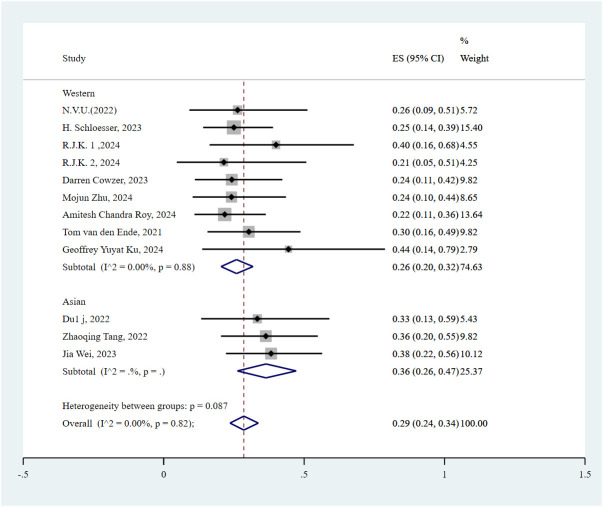
Forest plots of regional origin based on pathological complete response rate.

Stratification by geographic region identified pCR rates of 36% (95% CI: 26%–47%; I²=0%, P>0.10) in Asian cohorts and 26% (95% CI: 20%–32%; I²=0%, P = 0.88) in Western cohorts (**Figure 11**).

### 3.5 Publication bias

No significant publication bias was detected via Egger’s test regarding pCR rates (P = 0.160), MPR rates (P = 0.320), or the incidence of gradegrade ≥3 TRAEs (P = 0.742) (**Supplementary Figures 1**–**3**). It was indicated by sensitivity analysis that the overall results of this meta-analysis were not significantly influenced by any single study, thereby confirming the robust stability of these findings (**Supplementary Figures 4, 5**).

## 4 Discussion

This is the most comprehensive meta-analysis to date derived exclusively from prospective clinical trials investigating the integration of immunotherapy with nCRT for EGJ/GC. The data point to a regimen that is both highly effective and clinically manageable. The 29% pCR rate observed here surpasses historical benchmarks, such as the 23% reported in the adenocarcinoma cohort of the CROSS trial and the 15.6% in the POET study, while also outperforming the 20.3% rate seen in the TOPGEAR trial (2, 16, 20, 21), highlighting the potential benefit of adding PD-1/PD-L1 inhibitors. Regarding tumor regression, the observed 52% MPR markedly outperforms the historical 43% associated with traditional nCRT, suggesting a significant boost in tumor eradication through immunomodulation. Notably, our regimen indicates unique advantages in very high-risk populations, even when compared with emerging neoadjuvant chemo-immunotherapy protocols. Although the NEOSUMMIT-01 and -03 trials reported pCR rates of approximately 22%–28% for nICT, our study achieved a 29% pCR rate and 100% R0 resection rate, despite including a higher proportion of cT4b patients (22, 23). This highlights the potent role of radiotherapy in downsizing and converting locally advanced lesions. Specifically, for bulky or invasive lesions (T4b) where drug penetration of chemo-immunotherapy is limited, radiotherapy offers superior local control potential. This provides a more reliable opportunity for radical surgery in patients otherwise at risk of R1/R2 resection. The heterogeneity in MPR observed in this study may stem from multiple differences in clinical mechanisms. From a biological perspective, different chemotherapy exert distinct remodeling effects on the immune microenvironment. For instance, 5-FU selectively depletes myeloid-derived suppressor cells (MDSCs) to alleviate immunosuppression. Its strong synergistic effect with PD-1 inhibitors mechanistically explains the fluctuations in MPR rates across different chemotherapy settings (24, 25). Moreover, differences in the molecular structures and binding affinities of various PD-1/PD-L1 inhibitors may influence the intensity of T-cell activation and the depth of tumor regression (26). Finally, the lack of uniformity in total radiation doses and variations in radiotherapy techniques among the included studies may be key factors contributing to the high MPR heterogeneity in this pooled analysis. Concurrently, this deep pathological response was further translated into clinical outcomes. The literature consistently shows that ypN0 status translates into significantly extended disease-free and overall survival, along with a lower risk of recurrence, compared to those who remain ypN+ after neoadjuvant therapy (27, 28). In the present analysis, this synergistic combination pushed the ypN0 rate to 73% and T-stage downstaging to 51%. Perhaps most striking is the 100% R0 resection rate—a clear improvement over the 92% reported in the CROSS trial’s nCRT arm (2). Such findings establish a robust foundation for high-quality surgery and imply that the interplay between ICIs and nCRT could transcend the current efficacy plateau. These findings provide a theoretical basis for the clinical application of nCRT+ICIs and warrant further in-depth investigation. Admittedly, most studies included in this analysis were non-randomized, single-arm, early-phase (phase I/II) clinical trials with small sample sizes. This inherently limits the strength of our findings.

In terms of survival outcomes, the 1-year and 2-year OS rates pooled in this meta-analysis reached 91% and 74%, respectively. This result was comparable to the Phase III MATTERHORN trial (durvalumab plus FLOT regimen, 2-year OS rate of 75.7%) (29). However, while survival benefits were equivalent, the pCR rate observed in this study (29%) was significantly superior to the MATTERHORN trial (19.2%); this discrepancy strongly demonstrates that introducing radiotherapy on the basis of chemo-immunotherapy is a potent driver of pCR. The failure to translate this significant pathologic advantage into a greater survival benefit may stem from dual differences in baseline staging and immunological characteristics between this study population and previous trials. Specifically, although NEOSUMMIT-01 trial reported slightly higher 3-year OS data (81%), they were strictly limited to cT3-4a stage patients; in contrast, this study included more high-risk cT4b patients, and this baseline prognostic bias largely diluted the survival benefit values (23). Furthermore, the proportion of PD-L1 negative (CPS < 1) patients in this study population was as high as 38%. Although direct numerical comparison with the low-expression subgroup in the MATTERHORN trial (TAP < 1%, accounting for 10%) is limited by methodological differences between CPS and TAP systems and a lack of biological equivalence, the overall trend shows that the proportion of the PD-L1 low-expression population in this study was notably higher (29). Given that PD-L1 expression is a core factor determining immunotherapy sensitivity and that T4b patients face higher risks of systemic progression, this unfavorable baseline distributions objectively weakened the overall survival potential in this study. Additionally, the improvement in overall survival may also be related to the application of postoperative adjuvant therapy. In the NEOSUMMIT-01 trial, 90.2% of surgical patients initiated adjuvant therapy, and 25.5% of patients further received maintenance therapy with toripalimab monotherapy for as long as 6 months after completing combined chemotherapy. This design of long-term immune exposure aims to persistently clear minimal residual lesions and reduce the risk of long-term recurrence (22). In contrast, nearly half of the patients in this study did not receive postoperative adjuvant therapy due to various reasons, which may have led to the failure to effectively suppress tumor recurrence, thereby limiting the conversion of pathologic response into a survival advantage. Therefore, the low coverage rate of postoperative adjuvant therapy and the lack of its consolidation cycles may be key factors for the suboptimal overall survival data in this study. Admittedly, the current conclusions are still limited by the nature of non-randomized single-arm studies and the constraints of indirect comparison. Therefore, the results obtained are exploratory, and future prospective, head-to-head randomized controlled trials are urgently needed to confirm whether the local response advantage induced by radiotherapy can ultimately be translated into long-term survival benefits across different T stages and PD-L1 expression backgrounds.

Although this study observed a slightly higher incidence of grade ≥3 TRAESs (47%), which may require certain clinical monitoring, this result is basically consistent with the grade ≥3 TRAES rates of previously reported nCRT (15%–43%) and neoadjuvant chemotherapy plus immunotherapy (30%–71.6%) regimens, therefore indicating that the strategy of nCRT+ICIs for locally advanced EGJ/GC did not increase synergistic toxicity, and overall patient tolerance remained good (2, 29, 30). In terms of treatment integrity, despite slightly higher toxicity, 93% of patients still successfully completed the full neoadjuvant course, proving good clinical safety, and this safety was further verified during the surgical phase—based on the CROSS and POET trials showing that grade ≥3 complications after traditional nCRT range from 15% to 30% (2, 21), comparing to this study (range: 10.3%–39.4%), it was found that grade ≥3 complications of nCRT+ICIs remained basically consistent with historical data, and did not show new immune-specific surgical risks. Previous studies pointed out that grade ≥3 postoperative complications after traditional nCRT mainly consist of routine issues such as pulmonary infection, abdominal infection, and anastomotic leakage, while our study suggests that the spectrum of postoperative complications after nCRT+ICIs remains within the traditional range, and has not fundamentally changed the surgical risk, ensuring safety at the surgical level. Meanwhile, the stability of surgical timing further supports this conclusion, in the included studies, only Neo-PLANET and MC1541 reported surgical delays due to toxicity, the remaining trials did not see such cases (15, 19), current data suggest these brief delays did not affect efficacy. Notably, grade ≥3 TRAESs in this study were mainly concentrated in myelosuppression (e.g.: Cowzer reported a grade ≥3 TRAES incidence of 97%, 94% of which was attributed to lymphopenia) (8), which is a usually transient and clinically correctable hematologic event, therefore, the seemingly high incidence of grade ≥3 TRAESs is largely driven by asymptomatic laboratory abnormalities rather than treatment-limiting clinical toxicities, which explains the discrepancy between the high adverse reaction report rate and actual treatment compliance. The grade ≥3 irAEs rate of only 6% in this study provides a valuable reference. For comparison, the rate is approximately 3% for ICI monotherapy in advanced EGJ/GC (31) and 7%–8% for perioperative chemo-immunotherapy (e.g., KEYNOTE-585) (32). This suggests that the nCRT+ICIs strategy did not exacerbate synergistic toxicity. Subgroup analyses were performed to investigate the potential sources of grade ≥3 TRAES heterogeneity. Although PD-1 and PD-L1 inhibitors are pharmacologically distinct, their grade ≥3 TRAES incidences were nearly identical (47% vs. 48%), effectively ruling out the type of ICI as a primary source of heterogeneity. In contrast, radiotherapy dose was identified as a key factor; compared with low-dose radiation (<45 Gy), high-dose radiation (≥45 Gy) was associated with a significantly higher risk of severe TRAESs. This suggests that cumulative radiation dose, rather than the specific type of ICI, is the primary driver of toxicity fluctuations, likely due to its impact on systemic toxicities such as myelosuppression. Differences in dose distribution between 3D-CRT and IMRT may also be a factor, highlighting the need for standardized radiotherapy techniques in future research. Although sensitivity analyses were performed, persistent heterogeneity was observed. We believe that acknowledging these inherent diversities offers a more nuanced interpretation of the results, reflecting the true complexity of clinical settings. With the widespread clinical application of immunotherapy, differences in clinical efficacy between PD-1 inhibitors and PD-L1 inhibitors in cancer treatment have become increasingly apparent. Evidence from a recent meta-analysis (33) underscores the clinical superiority of PD-1 inhibitors, which achieved improved OS (HR, 0.75) and PFS relative to PD-L1 inhibitors. This trend is mirrored in our subgroup analysis, where PD-1 inhibitors yielded a 33% pCR rate compared to 26% for the PD-L1 cohort. Such a divergence likely stems from the inherent mechanistic differences between these two classes. While chemoradiotherapy-induced PD-L1 upregulation on tumor cells may theoretically enhance synergism, excessive PD-L1 expression could conversely counteract T-cell activation by saturating PD-L1 antibodies. PD-1 inhibitors, by contrast, retain a mechanistic edge by targeting the downstream receptor on T lymphocytes—a strategy that effectively circumvents this compensatory feedback (34). Crucially, PD-1 blockade simultaneously disrupts both the PD-1/PD-L1 and PD-1/PD-L2 axes, unlike PD-L1 inhibitors which are restricted to the former. Tumors may exploit the PD-1/PD-L2 pathway to evade immune surveillance during PD-L1-targeted therapy (35). Given that GC patients often exhibit substantial PD-L2 expression (36), our findings reinforce the clinical rationale for favoring anti-PD-1 antibodies in this setting.From a safety perspective, the incidences of grade ≥3 TRAESs were comparable between the groups (47% vs. 49%), with no statistically significant variance. Pharmacologically, PD-L1 inhibitors are purported to carry a lower risk of irAEs (37); this benefit may be offset, however, by the ADCC and CDC effects retained by certain PD-L1 inhibitors. Furthermore, in high PD-L1-expressing tumors, these agents may promote PD-L1 binding to RGMB on alveolar macrophages—a mechanism linked to immune-mediated pneumonitis not observed with PD-1 blockade (38). Conversely, PD-1 inhibitors may heighten the risk of gastrointestinal toxicities by disrupting PD-L2 signaling (39). These mechanistic insights may provide valuable guidance for clinicians. Nevertheless, these comparisons mandate caution, as unmeasured confounding variables, such as variations in chemotherapy regimen and patient baselines, likely introduced bias into the estimates. While these findings provide a valuable therapeutic signal, they await definitive confirmation through prospective, head-to-head trials.

Patients were stratified into sequential (ICIs followed by RT) or concurrent (simultaneous RT and ICIs) cohorts based on the temporal relationship between immunotherapy and radiotherapy (RT). No consensus has yet emerged regarding the optimal scheduling of these interventions, as both strategies are bolstered by distinct biological rationales and clinical data. Findings from a phase III trial by McCall et al. indicated that PD-1 inhibition prior to RT induced more pronounced tumor shrinkage than post-RT administration (40). This clinical benefit may stem from the pre-radiation enrichment of immune effector cells within the tumor microenvironment (TME), which primes the tissue for more effective infiltration and subsequent activation. On the other hand, concurrent delivery may be superior, as evidenced by the post-therapeutic expansion of transitional and tissue-resident memory B cells (41). Preclinical data support this synergy, showing that concurrent PD-L1 blockade sensitizes tumors to ionizing radiation (42), while radiation conversely primes the tumor for subsequent PD-L1 inhibition (43). As a result, both sequencing strategies are grounded in robust theoretical and clinical frameworks. In the present analysis, sequential and concurrent regimens yielded identical pCR rates of 29% (p > 0.05), suggesting no clear advantage for either strategy in the aggregate. To account for potential variation driven by specific ICI targets, we performed a more granular stratified analysis. This revealed that concurrent PD-1 inhibition achieved a 36% pCR rate—markedly higher than the rates observed for concurrent PD-L1 (25%) or sequential PD-L1 (29%) regimens. Such target-specific discrepancies likely reflect variations in the intensity of immune activation and spatial cellular distribution across different timelines, ultimately dictating the magnitude of the synergistic effect. Beyond pathological response, the influence of these scheduling strategies on immune memory formation and long-term survival remains poorly understood. Ultimately, identifying the optimal sequencing for ICIs and RT necessitates validation through large-scale, randomized trials that incorporate stratification by ICI subtype.

PD-L1 expression stands as a primary determinant of ICI efficacy and a cornerstone biomarker for predicting treatment response. In contemporary clinical practice, the Combined Positive Score (CPS) remains the standard metric for quantifying PD-L1 expression. The CPS is derived by dividing the number of PD-L1-positive cells—encompassing tumor cells, lymphocytes, and macrophages—by the total count of viable tumor cells, multiplied by 100. Compared to tumor-only scoring, this approach offers a more holistic view of the immunological milieu within the tumor microenvironment (44). International guidelines recommend the following gastric cancer PD-L1 expression stratification: CPS ≥5 for high expression, CPS 1 to 5 for low expression, and CPS <1 for negative. Among the patients receiving nCRT+ICIs in this study, the pooled pCR rates were 22% for the CPS <1 subgroup, 23% for the CPS 1–5 subgroup, and 51% for the CPS≥5 subgroup. This study demonstrates that the efficacy of nCRT+ICIs significantly improves with increasing PD-L1 CPS. This phenomenon is likely closely related to the high PD-L1 expression state in the tumor microenvironment indicated by a high CPS value. Radiotherapy may further synergistically enhance the antitumor effect of immunotherapy through mechanisms such as releasing tumor antigens and promoting immune cell infiltration (45). PD-L1 expression has been confirmed to be associated with deeper muscularis propria invasion, the presence of lymph node metastasis, and an overall poorer prognosis, particularly in patients treated with nCRT. The expression of PD-L1 and other immune biomarkers may also change following radiotherapy, further underscoring the potential synergistic effect between radiotherapy and immunotherapy. Therefore, these data support the exploration of nCRT+ICIs and indicate that this combination therapy offers higher pathological response rates and survival benefits in patients with high CPS, providing a critical basis for identifying potential beneficiary populations.

Real-world data indicate that over 50% of Chinese patients with advanced gastric cancer exhibit a CPS <5 (46). Notably, subgroup analyses from pivotal phase III trials of immunotherapy in gastric cancer (such as CheckMate 649 and ORIENT-16) demonstrated that patients with CPS <5 did not derive significant survival benefit from immune checkpoint inhibitor therapy (47, 48), highlighting the urgent need to optimize treatment strategies for this PD-L1 low-expression population. The synergistic effect between radiotherapy and immunotherapy offers a potential breakthrough. Basic research has shown that radiotherapy can remodel anti-tumor immune responses through multiple mechanisms. Firstly, a key mechanism is radiotherapy-induced ICD, which stimulates the recruitment and differentiation of T lymphocytes, promoting their ability to recognize and effectively attack tumor cells (49). Another crucial mechanism involves the activation of the cGAS-STING signaling pathway, eliciting a type I interferon cascade response (50). Furthermore, radiotherapy can upregulate the expression of MHC class I molecules on tumor cell surfaces, increasing the infiltration of CD8+ and CD4+ T lymphocytes and their antigen recognition of tumor cells, thereby enhancing the host immune system’s capacity to identify and eliminate tumor cells (51, 52). In our study, encouraging pCR rates were observed in the PD-L1 low-expression subgroups (22% and 23% in the CPS <1 and CPS 1–5 subgroups, respectively). These rates are numerically higher than those reported in the CPS <10 subgroup of KEYNOTE-585 (12.3%), the CPS <5 subgroup of DANTE (18.2%), and the CPS <5 subgroup of MATTERHORN (14%) (29, 32, 53), suggesting the potential of nCRT+ICIs for PD-L1 low-expression patients. Given that the study population had low PD-L1 CPS status—a characteristic previously associated with predicted lower response to immunotherapy—the promising pCR rates observed in our study can be viewed as evidence for a strategy converting so-called immunologically “cold” tumors into “hot” tumors when ICIs are combined with radiotherapy. This strategic advantage suggests that the synergy of chemoradiotherapy and immunotherapy may circumvent the treatment resistance traditionally linked to low PD-L1 expression. This combination strategy provides new therapeutic possibilities for patients with low PD-L1 expression. Future efforts should focus on expanding sample sizes and conducting mechanistic studies to further validate the generalizability of this phenomenon and to explore other potential biomarkers for optimizing personalized treatment choices for patients with low PD-L1 expression.

Evidence suggests that radiotherapy-induced immune modulation is likely governed by a dose-response relationship (54). Varying radiation doses recalibrate the immunological landscape through divergent mechanisms: high-dose delivery induces direct cytolysis, liberating a surge of tumor antigens for immune recognition; intermediate doses trigger the STING-interferon signaling axis, effectively activating a cellular “suicide switch” (55); conversely, low-dose radiation can transform “immune deserts” into “hot” microenvironments by promoting vascular normalization and shifting macrophage polarization (56). Based on these mechanisms, we hypothesized that the synergy between immunotherapy and radiotherapy would vary across dose levels. Our data demonstrated that the pCR rate reached 33% in the ≥45 Gy radiotherapy dose group combined with immunotherapy, showing an improvement compared to the <45 Gy group (26%). This may be attributed to high-dose radiotherapy’s ability to directly disrupt cancer cell DNA structure, inducing cell death. Furthermore, high-dose radiotherapy can activate immune cells *in vivo*, enhancing their activity and cytotoxicity against cancer cells. This activation of immune responses helps amplify the therapeutic effect beyond the directly irradiated cancer cells. Following high-dose radiotherapy, tumor-specific memory immune cells may be generated and persist long after treatment completion, enabling more effective attacks on recurrent tumors. However, dose-dependent toxicities warrant clinical vigilance. Our pooled analysis revealed that the incidence of grade ≥3 TRAESs was higher in the ≥45 Gy group (61%) compared to the <45 Gy group (32%) across different clinical trials. This result exhibited significant heterogeneity, likely influenced by factors such as target volume, the choice of immunotherapy agents, and the dosage and cycle number of concomitant chemotherapy regimens. Available data confirm that the addition of ICIs may not significantly compromise overall treatment compliance, and most toxicities can be effectively managed with clinical interventions. Future research should focus on analyzing correlations between dose-volume histogram parameters and immune-related toxicities to establish predictive models integrating radiotherapy dose, target volume, and toxicity risks, ultimately achieving a precise balance between efficacy and safety.

Subgroup analysis revealed a significant difference in pCR rates between Asian (36%) and Western (26%) populations receiving nCRT+ICIs. This regional disparity mirrors clinical observations in other malignancies, including small cell lung cancer and head and neck squamous cell carcinoma (57, 58). Studies indicate that proximal tumors are more prevalent in Western patients, whereas distal tumors and a higher proportion of intestinal-type tumors (Lauren classification) dominate in Asian populations (59, 60). This combination of specific anatomical location and more immunogenic histological subtypes may directly promote a more robust anti-tumor response triggered by immunotherapy, leading to superior efficacy. At the molecular level, ethnicity-specific mutation patterns in key driver genes (e.g., APC, ARID1A) may enhance immune recognition by increasing tumor mutational burden (TMB) in Asian populations, thereby mediating better treatment responses (61). Simultaneously, differing dietary habits may influence gut microbiota composition, potentially impacting immunotherapy efficacy in gastrointestinal cancers (62, 63). Beyond biological factors, variations in clinical staging and treatment protocols significantly contribute to regional pCR disparities. However, the mechanisms underlying these regional differences require further investigation. There may be a potential link between HER2 status and immunotherapy response in gastric cancer, as HER2 overexpression or mutation often drives tumors from “hot” to “cold” (64). Nevertheless, the KEYNOTE-811 study demonstrated that adding pembrolizumab to trastuzumab and chemotherapy results in a synergistic sensitization effect (65). However, this immune benefit is clearly PD-L1 dependent: compared to the targeted-plus-chemotherapy group, patients with CPS ≥1 in the combination immunotherapy group showed significant survival benefits, while those with CPS <1 did not. This indicates that PD-L1 CPS ≥1 remains a critical indicator for immunotherapy benefit in HER2-positive GC patients. Furthermore, combined with PANACEA study results, many scholars believe that PD-L1 expression may be the core predictor of immunotherapy efficacy regardless of HER2 status (66). Consequently, the clinical trials included in this study may focus more on CPS or PD-L1 expression with limited descriptions of HER2, which might hinder deeper stratified analysis. This also highlights the urgent need for standardized molecular biomarker reporting and improved clinical management in future research. Additionally, since squamous cell carcinoma accounted for only 1.4% (6 cases) of the study population, the current data cannot assess substantial differences in efficacy between adenocarcinoma and SCC subtypes. Therefore, our findings primarily apply to the management of GC and EGJ adenocarcinoma; the generalizability of this strategy to squamous cell histology remains to be elucidated.

This systematic review has several limitations. Primarily, the predominance of small-sample, single-arm, phase I/II trials inherently constrain the certainty of the evidence and the generalizability of our findings. The absence of parallel control groups makes it challenging to disentangle true therapeutic effects from potential confounding factors. Furthermore, early-phase trials tend to overestimate efficacy, often due to publication bias favoring positive results and stringent inclusion criteria that select for patients with fewer comorbidities. Moreover, small sample sizes are inherently susceptible to statistical fluctuations; a few exceptional responders can disproportionately inflate overall response rates, thereby limiting the generalizability of the conclusions. Crucially, nearly half of the pooled data originated from conference abstracts or interim reports. Integrating interim or incomplete data may overstate therapeutic outcomes due to attrition bias and significance-driven reporting. While providing timely clinical insights, these results remain preliminary; our estimates may be refined as more mature follow-up data emerge from these ongoing trials. Esophageal cancer, EGJ cancer, and GC possess distinct biological characteristics, and their treatment responses may vary accordingly. However, the available data are insufficient to support a definitive comparison based on anatomical location. These constraints notwithstanding, our analysis suggests that integrating PD-1/PD-L1 inhibitors into nCRT regimens potentially enhances outcomes for patients with EGJ or gastric cancer.

## 5 Conclusion

nCRT+ICIs exhibited substantial clinical value in locally advanced EGJ/GC, significantly improving pCR and MPR rates while inducing marked T/N downstaging with a manageable overall safety profile. Subgroup analyses identified PD-L1 CPS ≥ 5, Asian ethnicity, radiation dose ≥ 45 Gy, and concurrent PD-1 inhibitor use as potential predictors of superior pathological response. Notably, populations with low PD-L1 expression also derived benefit from the biological synergy of this combined regimen. Nevertheless, as current evidence stems primarily from Phase II or single-arm studies, these conclusions remain exploratory. The definitive clinical benefits necessitate further confirmation through upcoming large-scale, randomized controlled trials.

## Data Availability

The datasets presented in this study can be found in online repositories. The names of the repository/repositories and accession number(s) can be found in the article/**Supplementary Material**.
