# Supplementary material for: Efficacy and safety of neoadjuvant chemoradiotherapy combined with immunotherapy for locally advanced esophagogastric junction or gastric cancer: a systematic review and meta analysis
Source: Front Immunol. 2026 May 29;17:1745356. doi: 10.3389/fimmu.2026.1745356 (PMC13260261; doi:10.3389/fimmu.2026.1745356)
Supplement: Supplementary file 2 [file DataSheet1.docx]

Supplementary Material

Supplementary Figures


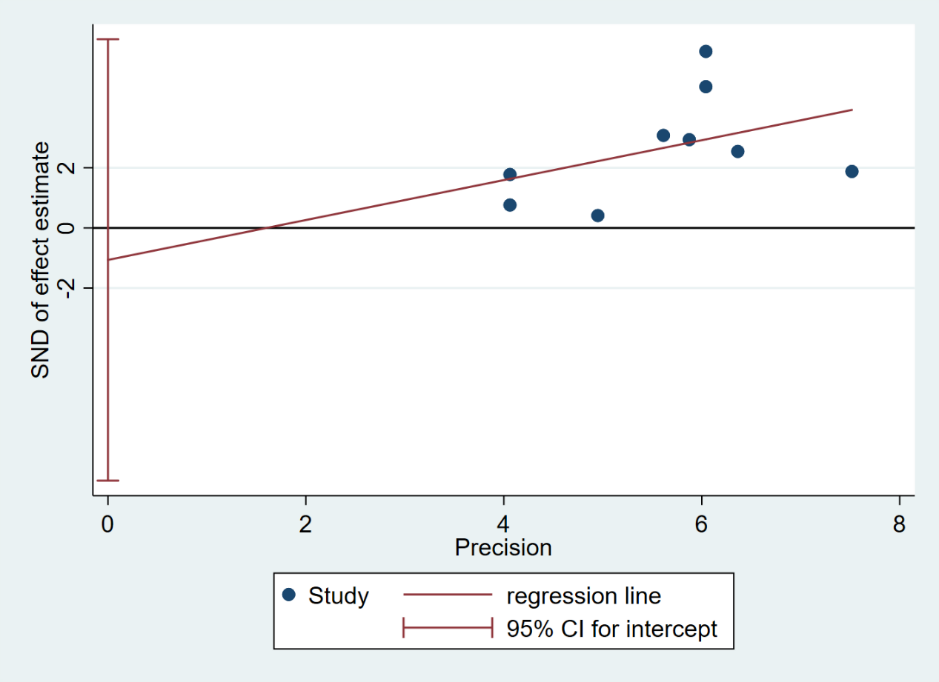


**Supplementary Figure 1.** Egger’s test of pathological complete response rate.
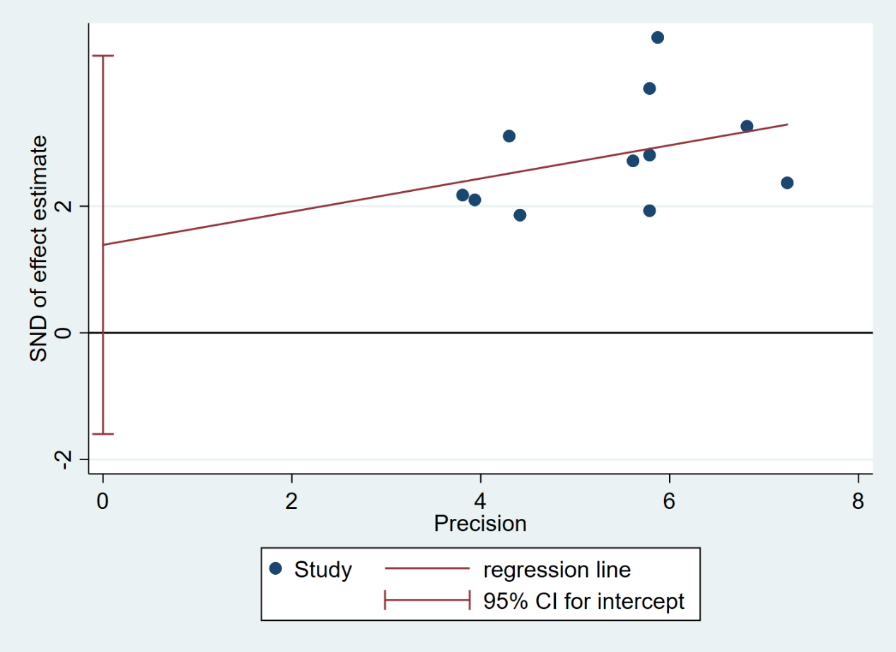
:

**Supplementary Figure 2.** Egger’s test of major pathological response rate.


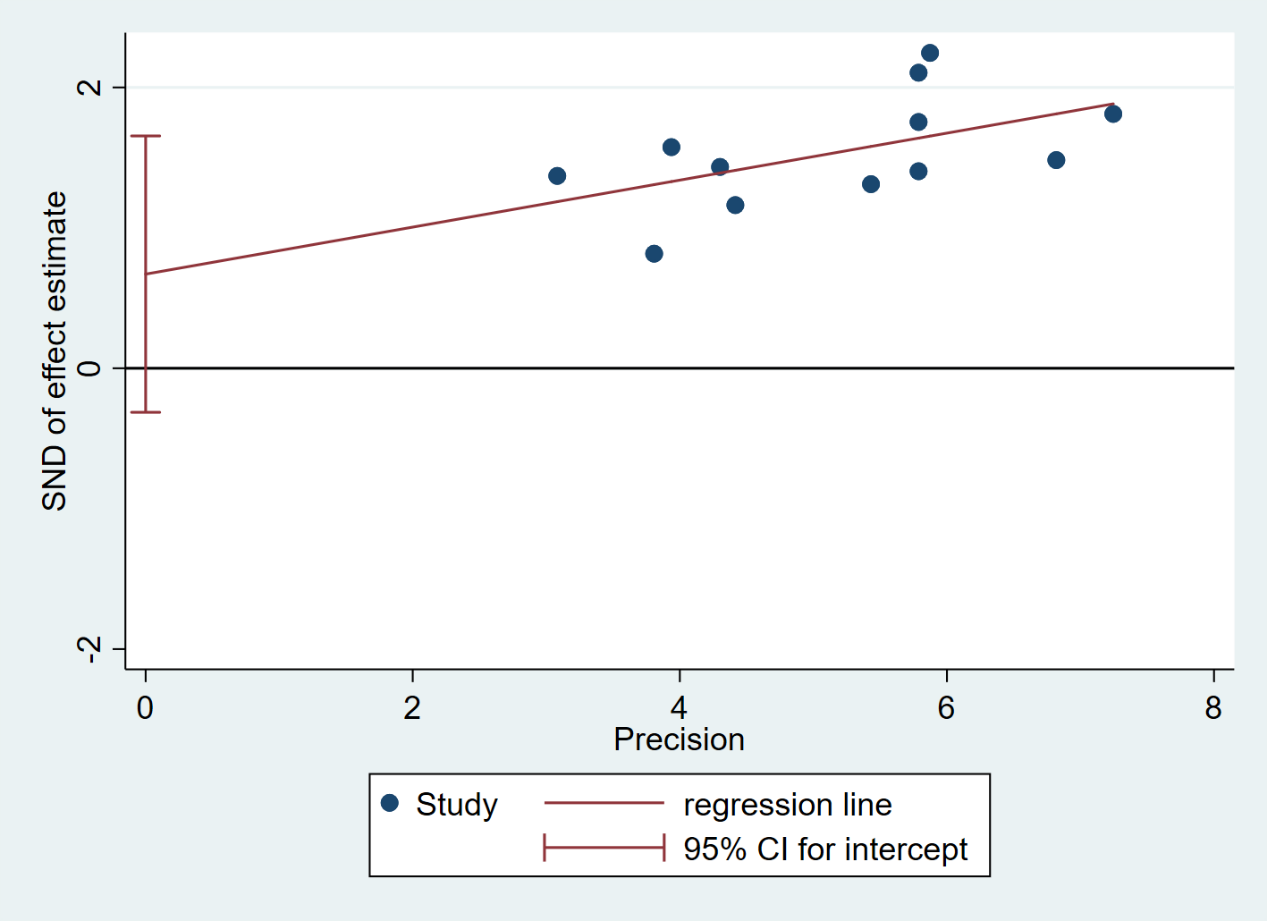


**Supplementary Figure 3.**Egger’s test of grade≥3 TRAEs


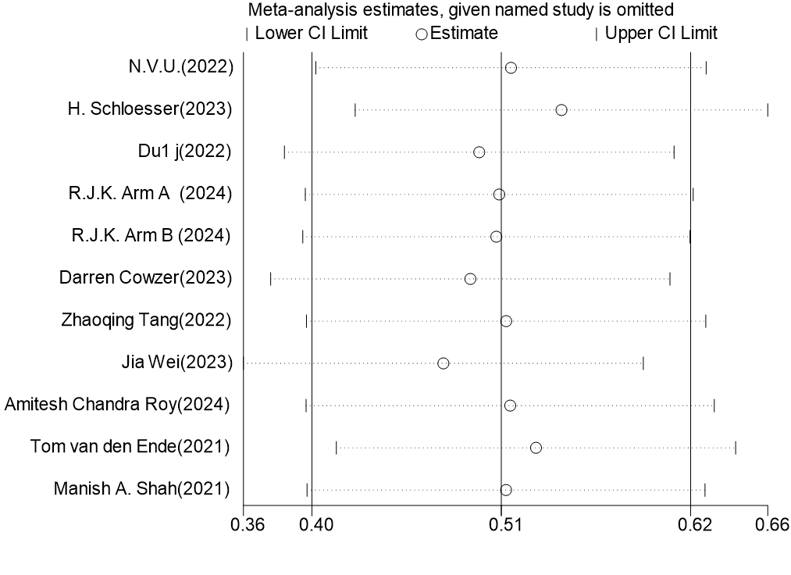
 **Supplementary Figure 4.** Sensitivity analysis of grade≥3 TRAEs.
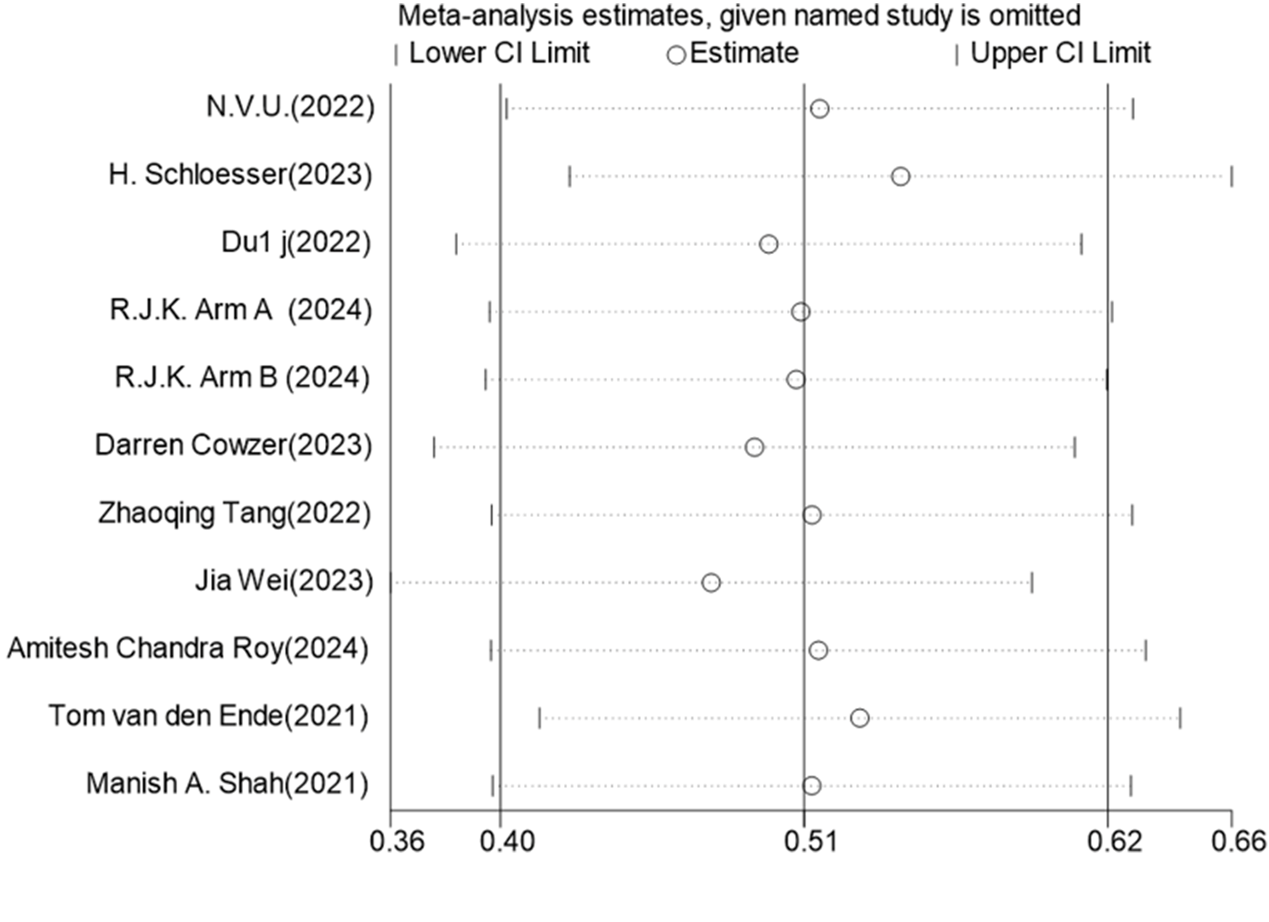


**Supplementary Figure 5.** Sensitivity analysis of major pathological response rate.
